# Natural killer cells and pigment epithelial-derived factor control the infiltrative and nodular growth of hepatic metastases in an Orthotopic murine model of ocular melanoma

**DOI:** 10.1186/s12885-019-5712-3

**Published:** 2019-05-22

**Authors:** Nyasia M. Jones, Hua Yang, Qing Zhang, Vanessa M. Morales-Tirado, Hans E. Grossniklaus

**Affiliations:** 1 Graduate Division of Biological and Biomedical Sciences Cancer Biology and Translational Oncology, Emory University, Atlanta, GA 30322 USA; 2 Department of Ophthalmology, Emory University School of Medicine, Atlanta, GA 30322 USA; 3 Department of Ophthalmology, Hamilton Eye Institute, University of Tennessee Health Science Center, Memphis, TN 38163 USA; 4 Department of Microbiology, Immunology, and Biochemistry, University of Tennessee Health Science Center, Memphis, TN 38163 USA; 5 Winship Cancer Institute at Emory University, 1365 Clifton Road NE, BT428, Atlanta, GA 30322 USA

**Keywords:** Hepatic metastases, Ocular melanoma, Tumor dissemination, NK cells, PEDF, Animal models

## Abstract

**Background:**

Metastases account for 90% of all cancer-related deaths, becoming a therapeutic problem. Approximately 50% of all uveal melanoma (UM) patients will develop metastases, mainly in the liver. *Post-mortem* analyses of livers from metastatic UM patients showed two different metastatic growth patterns: infiltrative and nodular. The infiltrative pattern exhibits tumor infiltration directly to the hepatic lobule and minimal angiogenesis. The nodular pattern shows clusters of tumor cells around the portal venules that efface the liver parenchyma. We recently demonstrated Natural Killer (NK) cells play a pivotal role in the control of hepatic metastases and the pigment epithelial-derived factor (PEDF) controls angiogenesis in the liver using our established ocular melanoma animal model. In this study we investigated the role of NK cells and PEDF in the development of metastatic growth patterns, as this can contribute to the development of novel therapeutics specific towards each growth pattern.

**Methods:**

We utilize our established ocular melanoma animal model by inoculation of B16-LS9 melanoma cells into C57BL/6 J mice (WT), anti-asialo GM1-treated C57BL/6 J mice (NK-depleted), and *PEDF*^*−/−*^ C57BL/6 J mice. Three weeks after inoculation we evaluated the metastatic growth patterns and stratified them based of the numbers of tumor cells. To evaluate angiogenesis the mean vascular density (MVD) was calculated. The immune compartment of the liver was analyzed by flow cytometry.

**Results:**

Our in vivo work showed two distinct metastatic growth patterns, the infiltrative and nodular, recapitulating the *post-mortem* analyses on human liver tissue. We discovered NK cells control the infiltrative growth. In contrast, PEDF controlled anti-angiogenic responses, showing higher MVD values compared to NK-depleted and WT animals. The myeloid lineage, comprised of monocytes, macrophages, and myeloid-derived suppressor cells, was reduced in the absence of NK cells or PEDF.

**Conclusions:**

Our animal model recapitulates the metastatic growth patterns observed in the human disease. We demonstrated a role for NK cells in the development of the infiltrative growth pattern, and a role for PEDF in the development of the nodular pattern. The understanding of the complexity associated with the metastatic progression has profound clinical implications in the diagnostic and disease-management as we can develop and direct more effective therapies.

## Background

Uveal Melanoma (UM) is the most common intraocular malignancy in adults [1, 2]. Despite effective control of the primary tumor, about 50% of UM patients develop metastatic disease. The mortality rate of these patients has not significantly changed in the last four decades due to the lack of an effective clinical treatment against metastatic disease [2]. The liver is the main site of primary metastasis in over 75% of cases, as UM tumor cells disseminate hematogenously [1, 3], and the CXCR4 [4–6] and c-Met [7, 8] receptors, which are present in UM, mediate migration toward ligand gradients produced by the liver. Studies aiming to understand the mechanisms and patterns of UM dissemination within the liver are limited. We recently identified two types of metastatic growth pattern through *post-mortem* analyses in human livers of metastatic UM patients: nodular and infiltrative [9]. The nodular pattern is characterized for growing adjacent to the portal venule effacing the surrounding hepatic parenchyma. The hepatocytes are pushed aside destroying the pre-existing liver architecture. These hepatocytes are separated from the tumor cells by a thin layer of reticulin fibers. In contrast, the infiltrative pattern shows invasion of the hepatic lobule, replacing healthy hepatocytes [9]. The metastatic cells invade the liver parenchyma without disturbing the pre-existing liver structure at the interface [10].

The landscape of metastatic progression is of great importance for the understanding of the interplay between tumor cells and the microenvironment. Niederkorn and colleagues [11] initially reported NK cell activity in the eye promoted the growth of UM. A follow up in vitro study by these authors suggested macrophage migration inhibitory factor (MIF) production by UM cells protects against NK cell-mediated killing [12]. Our group developed an ocular melanoma murine model and we formally demonstrated NK cells are pivotal for the control of hepatic metastases [13].

As the role of PEDF in suppression of ocular neovascularization was elucidated, our group discovered that the ratio of vascular endothelial growth factor (VEGF) to PEDF played a role in the migration of UM cells and hepatic metastases [14]. Furthermore, we recently demonstrated the role of PEDF as an anti-angiogenic and anti-stromagenic factor in UM [15]. Still, the role on NK cells and PEDF in the development of metastatic growth patterns and immune polarization is not understood. To evaluate the roles of NK cells and PEDF in the tumor microenvironment in metastatic UM in the liver, we compared the tumor microenvironment relative to metastatic UM growth using our established orthotopic murine model of ocular melanoma. Our results suggest a role for NK cells in the development of the infiltrative metastatic growth pattern and a role for PEDF in the nodular growth. We measured a reduction in the myeloid lineage within the metastatic liver and discovered the expression of both pro-inflammatory and anti-inflammatory genes.

## Methods

### Tumor and cell culture conditions

The mouse melanoma cell line B16-LS9 was kindly provided by Dario Rusciano at the Friedrich Miescher Institut, Basel, Switzerland. The complete culture medium included RPMI1640 with HEPES, L-glutamine, 10% FBS, 1% nonessential amino acids, 1% sodium pyruvate solution, 1% MEM vitamin solution, and a 1% antibiotic-antimycotic solution and incubated at 37 °C/5%CO_2_. Cells were grown to 90% confluence prior to harvest, washed with Hank’s solution, trypsinized and expanded for experiment use.

### Mice

Eight-week old female C57BL/6 J mice were purchased from Jackson Laboratories (Bar Harbour, ME). *PEDF*^*−/−*^ mice were generated on a C57BL/6 background as previously described [16] and provided courtesy of Dr. Susan Crawford (St. Louis University, St. Louis, MO). All experiments were performed according to the Declaration of Helsinki, in compliance with the Association for Research in Vision and Ophthalmology (ARVO) Statement for the use of animals in Ophthalmic and Visual Research and with the Institutional Animal Care and Use Committee policies and procedures from Emory University.

### Anti-Asialo GM1-mediated NK cell depletion

A cohort of 15 female C57BL/6 J mice was treated with anti-asialo GM1 serum to deplete NK cells. Anti-asialo GM1 (Rabbit; Wako Pure Chemical Industries, Osaka, Japan) was diluted to 1:4 in distilled water prior to injection. To achieve NK cell depletion mice were injected intraperitoneally (i.p.) with 100 μL anti-asialo GM1 every 3 days, beginning two weeks prior to cell line inoculation until they were euthanized [17].

### Murine model of ocular melanoma

Eight-week old female C57BL/6 J, C57BL/6 *PEDF*^−/−^, and C57BL/6 J anti-asialo GM1-treated mice were inoculated in the posterior compartment of the right eye with 5 × 10^5^ B16-LS9 melanoma cells in a 2.5 μL final volume using a transcorneal technique with a 30-gauge needle under guidance of a dissection microscope. The mice were anesthetized by intraperitoneal injection (i.p.) of 100 mg/kg ketamine and 12 mg/kg xylazine mixture in PBS. The inoculated eyes were enucleated after one-week. Mice were euthanized in a CO_2_ chamber 28 days post inoculation. Liver tissue were collected and processed for histological and cellular evaluation.

### Histology assessment

Excised livers were grossly examined and submerged in 4% neutral buffered formaldehyde for routine processing. Formalin fixed-paraffin embedded slides were stained with hematoxylin and eosin (H&E) and microscopically evaluated (Olympus, Waltham, MA) for metastases. Three sections through the center of each liver were evaluated for the number of metastases, and the average number per section was determined, as previously described [17, 18]. To determine relative metastasis area the sizes of all metastases in a single liver section were added to calculate a total metastasis area (μm^2^) and averaging this value per mouse. Metastases were separated by size into stage 1 (< 50 μm in diameter), stage 2 (50–200 μm), and stage 3 macrometastases (> 200 μm). Measurements were performed in triplicate and averaged per mouse as previously described [19].

### Evaluation of tumor vascular density

The number of blood vessels per 40x high-powered field (HPF) was calculated and averaged to mean vascular density (MVD). An individual vessel was defined as an area of lumen lined with endothelial cells, while tracts and branches were counted as separate vessels. C57BL/6 J, *n* = 9; C57BL/6 *PEDF*^−/−^, n = 9; and C57BL/6 J anti-asialo GM1-treated mice, *n* = 8.

### Cell suspension from liver tissue

Murine livers were isolated by maceration using a pestle (MidSci, St. Louis, MO) on a 70 μm nylon strainer (BD Biosciences, San Jose, CA). Cell suspension was washed and red blood cell contaminants were eliminated using 1x RBC Lysis Buffer (BioLegend, San Diego, CA) following manufacturers instructions. Cells were resuspended in PBS/1% FBS and kept on ice until ready to use.

### Cell surface labeling for flow cytometry analyses

Cell suspension was labeled with the following antibodies: anti-CD11b (clone M1–70) PE, anti-Ly-6C (clone HK1.4) FITC, anti-Ly-6G (clone 18A) APC, anti-Gr-1 (clone RB6-8C5) FITC, and anti-F4/80 (clone BM8) PE. All anti-mouse antibodies were purchased from BioLegend. We labeled each sample with 1 μg of each antibody and incubated on ice for 30 min in dark. Samples were washed 3x in PBS/1% FBS. Acquisition strategy: CD11b^+^ cells (myeloid lineage): we gated FSC versus SSC to eliminate debris (Gate 1), followed by plotting of CD11b versus SSC to acquire CD11b^+^ cells; CD11b-subsets: cells from Gate 1 were plotted CD11b versus Ly6C, or Ly6G, or Gr-1; Kupffer cells: F4/80 versus SSC. Immunopositivity was determined by using isotype controls of each antibody and unlabeled samples. Single label controls were set up using The AbC™ Total Antibody Compensation Bead Kit (Thermo Fisher Scientific, Waltham, MA). Data acquisition was done in BD Biosciences LSRII Flow Cytometer and data analyses performed using FlowJo vX.0.0.6 (FlowJo, LLC, Ashland, OR), as before [20].

### Gene expression analyses

RNA was extracted from cells or tissue using RNeasy Mini Kit (Qiagen Inc., Valencia, CA) following manufacturer’s conditions. We used 100 ng of RNA material for cDNA synthesis and further pre-amplification prior to assay set up to increase test sensitivity. Samples were run in Roche® LightCycler 480 and analyzed using the Comparative ∆C_T_ Method as before [21]. We used the following assays (Thermo Fisher Scientific): *Tnfa*: Mm00443258_m1; *Socs3*: Mm00545913_s1; *Arg1*: Mm00475988_m1; *Tgfb1*: Mm01178820_m1; *Gapdh*: Mm99999915_g1; *Casp3*: Mm01195085_m1; *Trp53*: Mm01731290_g1; *Cd68*:Mm03047340_m1.

### Statistical analysis

A one-way ANOVA or two-way ANOVA with Tukey’s post-test was performed to determine statistical significance. Experiments comparing the immune cell populations used The Holm-Sidak test. The value *p* < 0.05 was used to define statistical significance for all assays. All data was reported as mean ± SEM using Prism Graph Pad (GraphPad Software, La Jolla, CA).

## Results

### Increased number of hepatic metastases in the absence of NK cells and PEDF production

**Fig. 1:**
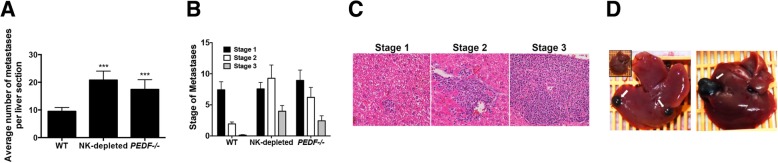
The absence of NK cells and PEDF increased the number of hepatic metastases. **a** Average number of metastases per liver section in an orthotopic murine model of ocular melanoma. NK cells were depleted from C57BL/6 J mice by treatment with anti-asialo GM1 serum; *PEDF*^*−/−*^ C57BL/6 mice were used for the *PEDF*^*−/−*^ orthotopic model. Data represents mean ± SEM, ***p < 0.001 using one-way ANOVA with Tukey’s post-test. Total mice per group: WT: *n* = 13, NK depleted: *n* = 17, *PEDF*^*−/−*^: *n* = 16. **b** Size of individual metastases were measured and stratified based on the following, stage 1: metastases < 50 μm; stage 2: metastases 50–200 μm; stage 3: macrometastases > 200 μm. Data represents mean ± SEM, statistical analyses done using The Holm-Sidak t-test; *p < 0.05, **p < 0.005, ***p < 0.0005. **c** Representative histological images of the different stages. **d** Grossly visible metastases from NK-depleted group. Metastases were visible to the naked eye following liver harvesting of NK-depleted mice, 3-weeks post inoculation of cells

We investigated the average number of hepatic metastases in our orthotopic model in the absence of NK cells and PEDF. The B16-LS9 melanoma cell line was inoculated into the posterior compartment of wild type C57BL/6 J mice (WT), C57BL/6 J mice treated with anti-asialo GM1 serum to deplete NK cells (NK-depleted) and *PEDF*^*−/−*^ C57BL/6 J mice (*PEDF*^*−/−*^). Hepatic metastases were counted after 3-weeks post inoculation and compared in all groups. We measured a significant increase in the average number of metastases in the NK-depleted (***p < 0.001) and *PEDF*^−/−^ (***p < 0.001) groups compared to WT controls (Fig. 1a). Next, we stratified the metastases based on a 3-staging system where stage 1 includes metastases < 50 μm in size, stage 2 is between 50 and 200 μm, and stage 3 comprised > 200 μm in size. As shown in Fig. 1b, WT livers showed a predominant stage 1 while NK depleted and *PEDF*^*−/−*^ groups had increased numbers of stage 2 and 3 metastases. A more detailed comparison between NK depleted and *PEDF*^*−/−*^ groups revealed a predominance of stage 2 in the NK depleted group and higher numbers of both stage 2 and 3 compared to the *PEDF*^*−/−*^ group. Representative images of the stages of the hepatic metastases are shown in Fig. 1c. The tumors were grossly visible upon examination, as shown in Fig. 1d.

### Inhibitory effect of PEDF on the nodular pattern of metastatic growth

**Fig. 2:**
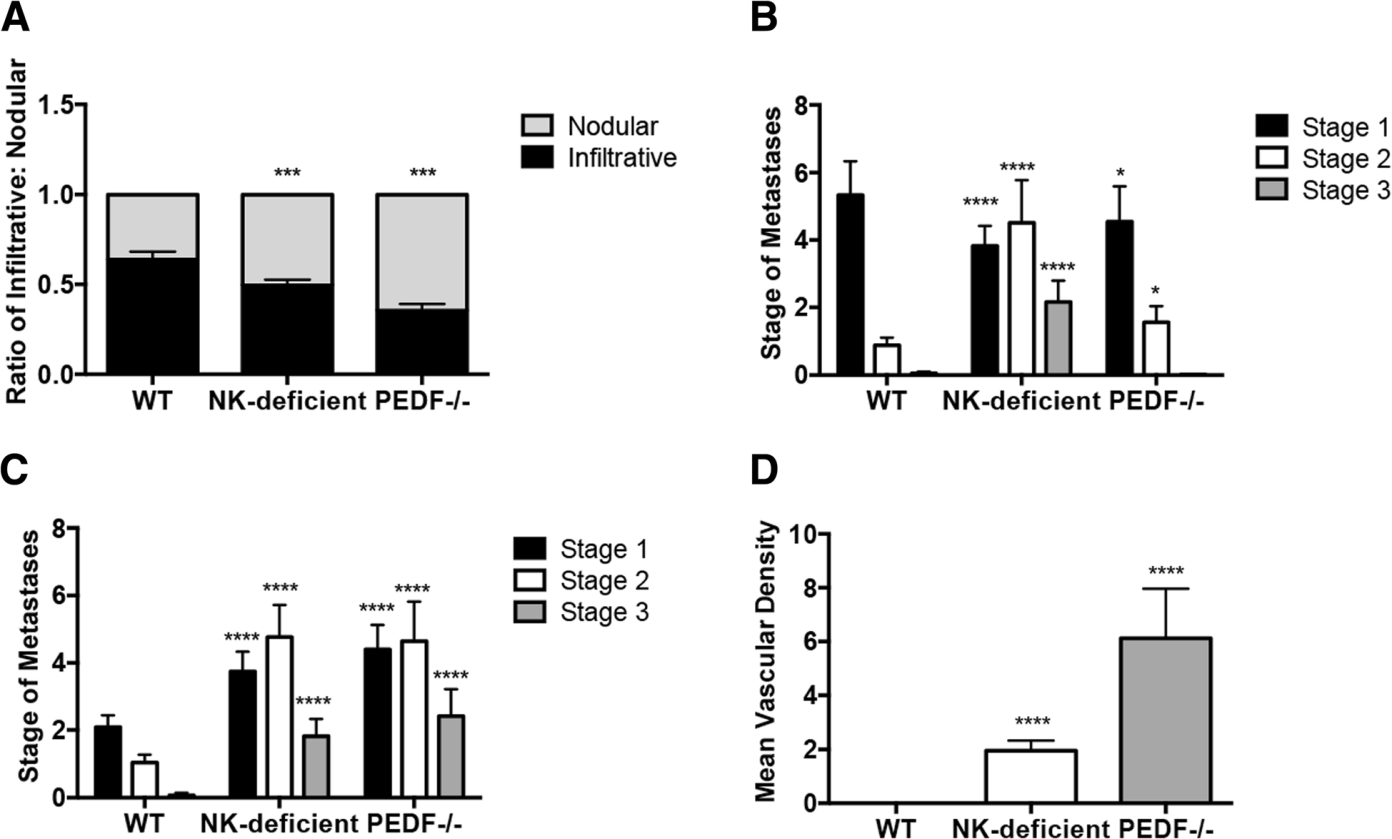
Increase in nodular growth in NK depleted and *PEDF*^*−/−*^ orthotopic grafts. **a** The ratio of infiltrative to nodular metastasis was determined from the average number of infiltrative and nodular metastases per mouse. These were then averaged for each group to establish an overall average for each experimental group. NK depleted and *PEDF*^*−/−*^ groups were compared to WT using a two-way ANOVA with Tukey’s post-test. Data represents mean ± SEM, ***p < 0.001. Total mice per group: WT: n = 13, NK depleted: n = 17, *PEDF*^*−/−*^: n = 16. **b** ***left***, NK-depleted group showed a significant increase in the number of stage 2 and 3 infiltrative metastases in comparison to WT and *PEDF*^*−/−*^ groups. *PEDF*^*−/−*^ group showed a significant increase in the average number of stage 2 infiltrative metastases. Data represents mean ± SEM, ****p < 0.0001 using two-way ANOVA with Tukey’s post-test. **b** ***right***, NK depleted and *PEDF*^*−/−*^ groups showed a significant increase in the average number of stages 2 and 3 nodular metastases compared to WT group. Data represents mean ± SEM, ****p < 0.0001, using two-way ANOVA with Tukey’s post-test. **c** Representative images of Stages 1–3 of the infiltrative and nodular metastatic growth patterns. Black arrow points at the micrometastases; white arrows depicts the metastatic cells adjacent the portal venule. **d** Increase in the mean vascular density in the metastatic tissue of NK depleted and *PEDF*^*−/−*^ groups compared to WT. MVD calculated as the average of the number of blood vessels per 40x HPF. An area of lumen lined with endothelial cells was considered an individual vessel; tracts and branches were counted as separate vessels. C57BL/6 J, *n* = 9 mice; C57BL/6 *PEDF*^−/−^, n = 9; and C57BL/6 J anti-asialo GM1-treated mice, *n* = 8. Data represents mean ± SEM, ****p < 0.0001, using one-way ANOVA with Tukey’s post-test

We sought to investigate the contribution of NK cells or PEDF in UM metastatic growth patterns (Fig. 2a) by measurement of the ratio of infiltrative: nodular growth. WT animals measured an infiltrative: nodular ratio of 3:1, similar to our *post-mortem* analyses on human livers from metastatic UM patients [9]. This ratio changed in the absence of NK cells towards an equal proportion of infiltrative and nodular growth patterns. In the absence of PEDF there was an increased ratio of nodular to infiltrative growth pattern compared to both WT and NK depleted groups (***p < 0.001). Next, we stratified the growth patterns based on the staging system described in Fig. 1. We measured a significant increase in the average number of metastases following the infiltrative growth pattern in stage 2 (*p < 0.05) and 3 (**p < 0.005) in the NK depleted group compared to WT (Fig. 2b, ***left***). The *PEDF*^*−/−*^ group showed a significant increase in the average number of metastases following the infiltrative patter in stage 2 (**p < 0.005) compared to WT. There was a significant increase in the examination of nodular growth across all stages in both NK depleted (stage 1: *p < 0.05; stage 2: **p < 0.005; stage 3: *p < 0.05) and *PEDF*^*−/−*^ (stages 1–3: *p < 0.05) groups compared to WT, as shown in Fig. 2b**,** ***right***. WT control showed no stage 3 metastases, confirming our findings shown in Fig. 1b. Representative images of each stage within the infiltrative or the nodular growth patterns is shown in Fig. 2c. The infiltrative growth pattern showed metastatic cells within the hepatic lobule (Fig. 2c**,** ***upper panels***). In contrast, the nodular growth pattern exhibited metastatic cells adjacent the portal venule (Fig. 2c**,** ***lower panels***), represented by the white arrow.

Mean vascular density (MVD) was investigated as an angiogenesis measurement suggested correlates with metastatic development. In Fig. 2d we compared the MVD of metastatic tissue by counting the number of blood vessels per 40x high power field of magnification (40HPF). Our results showed a significant increase in MVD in the NK depleted (****p < 0.00005) group compared to WT. The *PEDF*^*−/−*^ group (****p < 0.00005) showed a significant increase in MVD compared to NK depleted and to WT animals.

### NK cell depletion and PEDF inhibition are independently associated with a reduction in myeloid lineage

**Fig. 3:**
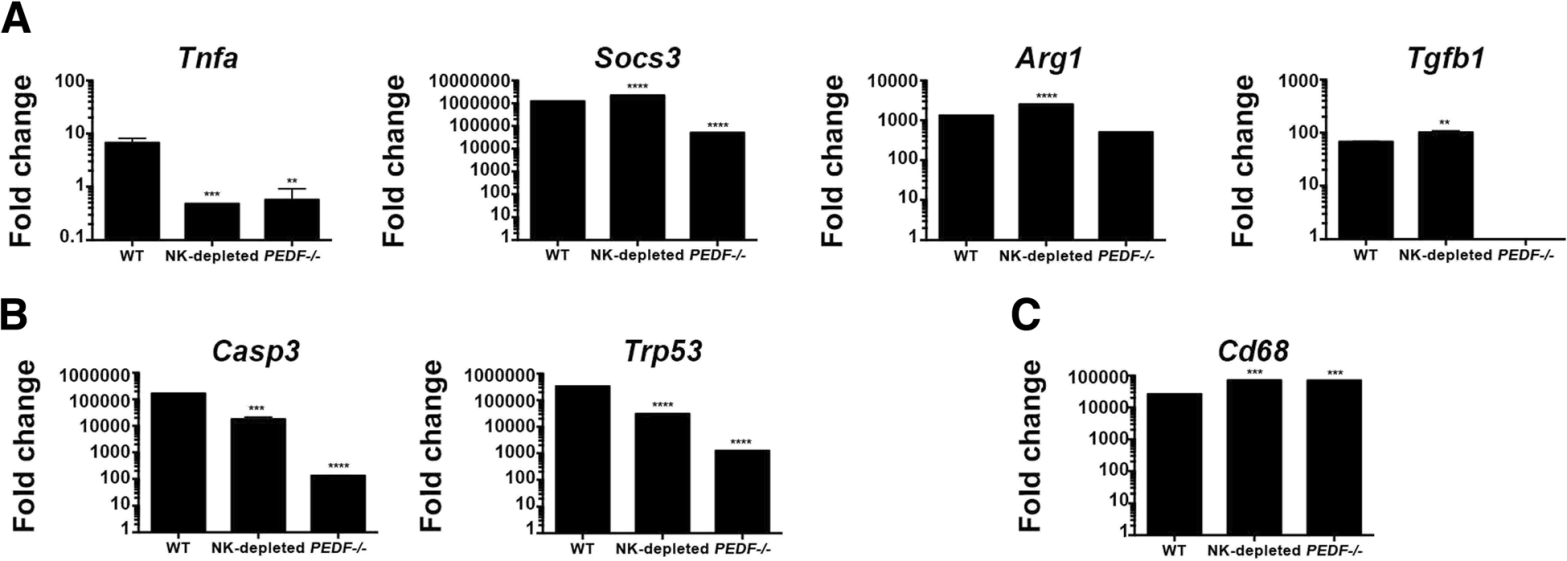
Reduction in the myeloid immune compartment in the absence of NK cells PEDF does not reduce hepatic metastases. **a-c** Excised livers from all groups were macerated for RNA extraction, cDNA synthesis and examination of mRNA expression. **a** Gene expression analyses of the type-1 genes *Socs3* and *Tnfa* mRNA and the type-2 *Arg1* and *Tgfb* mRNA using the ∆C_T_ Comparative method. **b** The pro-apoptotic Casp3 mRNA and the tumor suppressor Trp53 were examined using the ∆C_T_ Comparative method, as well. **c** Cd68 mRNA, typical of infiltrative macrophages, was examined in the bulk liver tissue. All Data represents mean ± SEM. All mRNA analyses display the fold change of target gene over endogenous control (*Gapdh*)

The interplay between the tumor cells and the microenvironment is of great importance for the understanding of the metastatic progression. We investigated if the absence of NK cells or PEDF in addition to favor a metastatic growth pattern, it may exhibit a polarized type of immune response. A number of markers have been identified for the M1 or pro-inflammatory macrophages and the M2 involved in tissue remodeling and immune regulation. We measured *Tnf* and *Socs3* mRNA expression by qPCR analyses to indicate M1 polarization in the liver tissue from all groups. Our results shown in Fig. 3a demonstrated higher expression of *Tnf* mRNA in WT compared to the other groups. There was 50% reduction in the NK depleted and *PEDF*^*−/−*^ groups compared to WT. Measurement of *Socs3* mRNA revealed higher expression in WT and NK depleted groups compared to the *PEDF*^*−/−*^ group. Next, we chose *Arg1* and *Tgfb* as genetic markers for M2 polarization. All groups measured *Arg1* mRNA, while only the WT and NK depleted groups measured *Tgfb* mRNA expression. The liver microenvironment showed mRNA expression of M1 and M2-type genes.

Next, we investigated the tumor suppressor mechanisms associated with the hepatic metastatic growth in the absence of NK cells and the absence of PEDF. Both *Casp3* and *Trp53* genes were downregulated in the NK-depleted and *PEDF*^*−/−*^ animals compared to WT. Moreover, the reduction in these genes was stronger in the absence of PEDF, as shown in Fig. 3b.

**Fig. 4:**
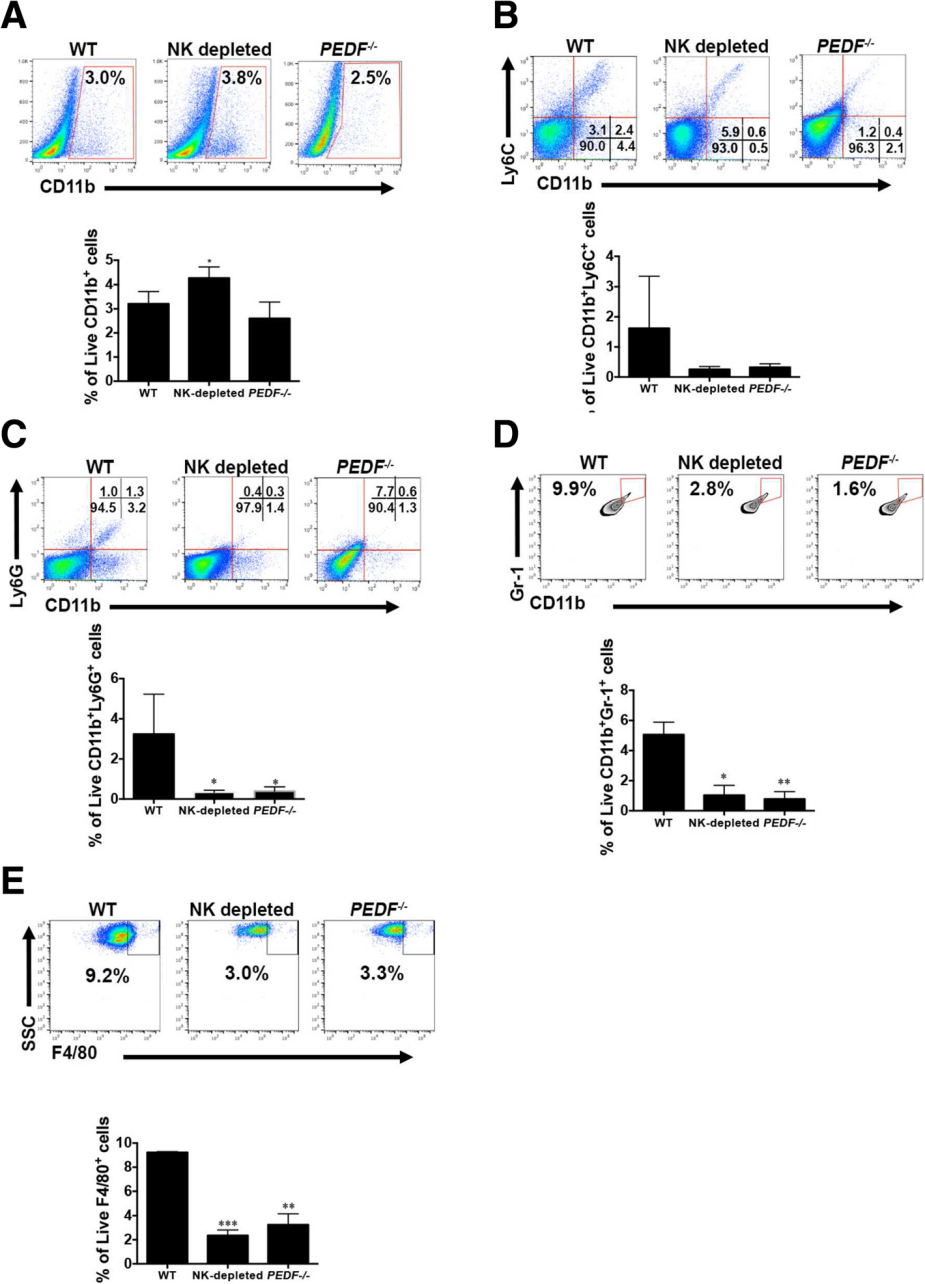
Percentages of live CD11b^+^, which comprise monocytes, neutrophils, dendritic cells, and some NK cells. **a-e** Another part of the livers was macerated followed by removal of red blood cells. Remaining cells were labeled with anti-CD11b^+^ followed by flow cytometry analyses. **a** ***upper panel***, Red gate indicates the percentage of CD1b^+^ cells. ***Lower panel*** shows bar graph summarizing the data. **b-e** The CD11b^+^ analyses were subdivided based on the presence of cell surface markers to determine the percentage of CD11b^+^Ly6C^+^ (**b**), CD11b^+^Ly6G^+^ (**c**), CD11b^+^Gr-1^+^ (**d**), and F4/80^+^ (**e**) cells. In Fig. 4**a-c,** and **d**, the ***upper panel*** shows a representative pseudocolor FACS plot from each of the groups and the ***lower panel*** shows a bar graph summarizing the data. Figure 3**d,** ***upper panel*** shows a Zebra plot for each of the groups; ***lower panel*** shows bar graph with data summary. All data is represented as mean ± SEM using The Holm-Sidak t-test; *p < 0.05, **p < 0.005, ***p < 0.0005

Our interest in the role of different myeloid populations in the clearance of hepatic metastases led us to first, investigate the gene expression of *CD68*, a biomarker of infiltrative monocytes, in the liver microenvironment. We measured high expression of *CD68* mRNA across all groups. The expression was higher in the NK-depleted and *PEDF*^*−/−*^ groups compared to WT (Fig. 3c). In Fig. 4a we measured the percentage of live CD11b^+^, which comprise monocytes, neutrophils, dendritic cells, and some NK cells [22–25], to investigate if the increase in hepatic metastases and angiogenic activity was due to modulation of this population. Figure 4a**,** ***lower panel*** showed a small increase in CD11b^+^ cells in the NK-depleted livers. Representative pseudocolor plots of each group are shown in Fig. 4a**,** ***upper panel***. Myeloid derived suppressor cells (MDSCs) have distinct phenotypes based on Ly6C or Ly6G expression. There was a striking reduction, albeit not significant, in the percentage of live CD11b^+^Ly6C^+^ cells in the NK-depleted and *PEDF*^*−/−*^ groups compared to WT (Fig. 4b). However, there was a significant reduction in the percentage of live CD11b^+^Ly6G^+^ cells, which are considered neutrophils, in the NK-depleted (*p < 0.05) and *PEDF*^*−/−*^ (*p < 0.05) groups compared to WT (Fig. 4c). Quantitation of cells with the immature myeloid phenotype CD11b^+^Gr-1^+^ (a composite epitope between the Ly6C and Ly6G antigens) revealed a significant reduction in the percentage of live CD11b^+^Gr-1^+^ cells in the NK depleted (*p < 0.05) and *PEDF*^*−/−*^ (**p < 0.005) groups compared to WT (Fig. 4d). The percentage of live Kupffer cells with a phenotype of F4/80^+^ cells was significantly reduced in the NK depleted (***p < 0.0005) and *PEDF*^*−/−*^ (**p < 0.005) groups compared to the WT (Fig. 4e). Collectively, our results showed depletion of NK cells or absence of PEDF reduces the percentages of MDSC and correlates with an increase in hepatic metastases.

## Discussion

Our findings suggest NK cells and PEDF play a role in the growth pattern of metastatic UM. Hepatic metastases are more abundant in the absence of NK cells and PEDF. Moreover, we characterized for the first time the growth patterns of metastatic UM in an orthotopic murine model of ocular melanoma. The WT group shows a higher ratio of infiltrative to nodular metastatic pattern similar to that observed in post mortem human livers from metastatic UM patients [9]. The ratio of infiltrative to nodular is reduced in the absence of NK cells and PEDF. Divergence among these two was observed in the studies characterizing the microenvironment and in the tumor vascular supply. NK cells play a role in the microenvironment of the hepatic metastases while PEDF is more critical for the angiogenesis.

We have previously demonstrated that the hepatic sinusoidal space contains resident NK cells [9]. However, these NK cells are in an immature state. Recent investigations from our group showed the Toll-like receptor-5 (TLR-5) agonist entolimod enhanced anti-metastatic activity against hepatic metastases by mobilization of NK cells to the liver, and stimulate the maturation, differentiation and activation of the resident ones [13]. Because of the presence of NK cells in the sinusoidal space within the infiltrative pattern of metastatic growth we hypothesized that depletion of this population will shift the ratio of infiltrative to nodular, increasing the nodular pattern. Our results supported our hypothesis as it increased the number of metastases following the nodular pattern. This increase in nodular pattern shifted the ratio to a 1:1, showing a similar distribution of infiltrative and nodular patterns. The staging or stratification of the hepatic metastases also differed in the absence of NK cells. In both the infiltrative and nodular patterns the hepatic metastases followed the patter of higher stage 2 > stage 1 > stage 3, compared to the WT group, which followed stage 1 > stage 2 > stage 3. Collectively, our work supports a critical role for NK cells in the control of hepatic metastases.

Multiple groups, including ours, have investigated M1/M2 polarization as predictors of outcome in UM patients [26–33]. A number of molecular markers and cytokines are used to classify macrophages into M1 and M2. Classically, M1 are considered the destroyers of tumor cells. These M1 macrophages present antigen to T cells, produce high levels of pro-inflammatory cytokines, and express both nitric oxide synthase and reactive oxygen species [34, 35]. In contrast, M2 suppress type-1 immune responses, promote tissue remodeling and wound healing, angiogenesis via VEGF and promote tumor development [34, 36]. We examined the genetic expression of two markers associated with M1 and M2 polarization. The absence of NK cells measured similar expression of *Socs3* mRNA to WT groups, associated with tumor killing, but base levels of *Tnf* mRNA, a key pro-inflammatory cytokine in anti-tumor responses. Similar *Arg1* and *Tgfb* mRNA expression were observed in the absence of NK cells compared to WT groups. This raises multiple questions for further investigations on the plasticity of these macrophages and their contribution in angiogenesis and disease progression [37].

Tumor cell survival is associated with a decreased capacity of tumor suppressors, such as p53, to execute their function. P53-dependent apoptosis is induced by activation of effector caspases, such as caspase-3 [38]. In our investigation we measured reduction of both *Casp3* and *Trp53* mRNA in NK-depleted animals compared to WT. This data confirms caspase-3 is an important effector molecule in NK cell-mediated apoptosis in tumors. The *PEDF*^*−/−*^ animals measured a stronger effect on downregulation of both *Casp3* and *Trp53* mRNA. Work from Takenaka and colleagues [39] recently demonstrated activation of caspase-3 and induction of apoptosis by PEDF using the MG63 human osteosarcoma cells. These results suggest PEDF, in addition to decreasing hepatic metastases by eliciting anti-angiogenic responses [15, 19] also regulates caspase-3 activation. Based on the work performed in the human livers from metastatic UM patients [9] we hypothesized that the nodular pattern is controlled by PEDF in the periportal area, thus, absence of PEDF will increase the ratio of nodular to infiltrative growth. Our results supported our hypothesis as the *PEDF*^*−/−*^ group had a significant increase in the metastatic growth following the nodular pattern compared to WT and NK depleted groups. Nodular metastases in human livers express MMP-9, VEGF, and contain CD31^+^ vascular channels [9]. Therefore, we can speculate PEDF from hepatocytes inhibit the VEGF from the tumor microenvironment. Here, we provide evidence of the anti-angiogenic role of PEDF in the hepatic metastases as the *PEDF*^*−/−*^ grafts showed a significant increase in MVD compared to WT.

Characterization of the myeloid-derived cells in the liver microenvironment suggests that dysfunction of multiple cells types can lead to failure to control hepatic metastases. Our results showed a reduction in CD11b^+^Ly6C^+^, CD11b^+^Ly6G^+^, and CD11b^+^Gr-1^+^ cells in the NK-depleted and *PEDF*^*−/−*^ groups. Both groups failed to control the growth of hepatic metastases in our orthotopic ocular melanoma model. These results are consistent with the concept that the absence of tissue infiltrating myeloid-derived cells modifies the tumor microenvironment, leading to failure to control tumorigenesis [40, 41]. Neutrophils are considered of great importance in the control or the progression of different cancers [42–44]. However, the dual roles of neutrophils, as an anti-tumor and pro-tumor functions, needs to be further studied [45–49]. Recent work from Sadegh et al. [50], demonstrated the development of hepatic metastases to be associated with the upregulation of IL-10 in the liver microenvironment and its receptor on NK cells. The source of IL-10 was confined to bone marrow-derived cells that are not MDSCs. Sadegh et al., used Gr-1-depletion to investigate if MDSCs were the source of IL-10. It has been demonstrated Gr-1^+^ is also a neutrophil marker; therefore, in our investigations we characterized cells based on Gr-1- and Ly6C/G-positivity. MDSCs have been considered pro-tumoral at the systemic level. Our work suggests that the presence of these cells have an anti-tumoral effect, as their percentages are reduced in those groups with elevated number of metastases. It is tempting to speculate their effector function is locally within the liver microenvironment.

In this investigation we demonstrated the two distinctive metastatic growth patterns in hepatic metastases of an orthotopic murine model of ocular melanoma. We demonstrated NK cells play a critical role in the control of hepatic metastases following an infiltrative growth pattern. In addition, we demonstrated the importance of PEDF in the control of angiogenesis in this animal model, which also contributes to the generation of large nodular growth. Together, our work provides a novel understanding of the infiltrative and nodular patters of metastases and on the role of myeloid-derived suppressor cells in metastatic UM.

## Conclusions

The majority of cancer mortality is associated with tumor dissemination rather than the primary tumor. The development of metastases is correlated with poor prognosis and there are limitations for effective anti-metastatic treatments. Herein, we provide a mechanistic understanding of the growth patterns associated with hepatic metastases, namely infiltrative and nodular, using an orthotopic murine model of ocular melanoma. Furthermore, we characterized the myeloid immune compartment in the tumor microenvironment by flow cytometry analyses. These analyses revealed a reduction in macrophages and myeloid-derived suppressor cells (MDSCs), which promoted an anti-inflammatory response in both NK cell deficient and *PEDF*^*−/−*^ groups, suggesting MDSC reduction does not confer a survival advantage. Our findings represent a novel understanding of the infiltrative and nodular patters of metastases and on the role of MDSCs in metastatic UM.

## Authors’ information

HY is an Associate Professor at the Research Section of the Department of Ophthalmology at Emory University, who focuses on anti-angiogenic and immune modulatory therapies against metastatic uveal melanoma. VMT is an Assistant Professor at the Departments of Ophthalmology and Microbiology, Immunology and Biochemistry (MIB) at the University of Tennessee Health Science Center. She leads the Ocular Oncology and Immunology Translational Research Unit at UTHSC and has expertise in human tumor immunology, intraocular tumors and pre-clinical models of disease. HEG serves as the Interim Vice Chair at the Emory Eye Center, founding director of the Ocular Oncology and Pathology Service and Director of the L.F. Montgomery Laboratory at Emory. HEG is considered an international expert in diagnostic ophthalmic pathology, and ocular oncology with over 500 publications in peer reviewed journals, over 25 book chapters and edited 10 books.

## Abbreviations

APC: Allophycocyanin
*Arg1*: Arginase 1 gene
*Casp3*: Caspase-3 gene
CD31: Cluster of differentiation 31
*CD68*: Cluster of differentiation 68 gene
cDNA: Complementary deoxyribonucleic acid
c-Met: Tyrosine-protein kinase Met
CXCR4: C-X-C chemokine receptor type 4
FBS: Fetal bovine serum
FITC: Fluorescein isothyocyanate
FSC: Forward scatter
H&E: Hematoxylin and eosin
HPF: High-powered field
i.p.: Intra-peritoneal
M1: Macrophage type-1
M2: Macrophage type-2
MDSC: Myeloid-derived suppressor cells
MEM: Minimum Essential Media
MIF: Macrophage migration inhibitory factor
mRNA: Messenger RNA
MVD: Mean vascular density
NK: Natural killer cells
P53: Tumor protein 53
PE: Phycoerythrin
PEDF: Pigment epithelial-derived factor
qPCR: Quantitative polymerase chain reaction
RNA: Ribonucleic aci
SEM: Standard error measurement
*Socs3*: Suppressor of cytokine signaling 3 gene
SSC: Side scatter
*Tgfb*: Transforming growth factor beta gene
TLR-5: Toll-like receptor 5
*Tnf*: Tumor necrosis factor alpha gene
*Trp53*: Transformation related protein 53 gene
UM: Uveal melanoma
VEGF: Vascular endothelial growth factor
WT: Wild type
